# Association between dipeptidyl peptidase-4 inhibitor use and diabetic retinopathy: a systematic review and meta-analysis of real-world studies

**DOI:** 10.1186/s12886-024-03535-1

**Published:** 2024-06-28

**Authors:** Minxi Wang, Jiali Lu, Jiyue Dong

**Affiliations:** Department of Ophthalmology, Changxing County People’s Hospital, 66 Taihu Middle Road, Zhicheng Town, Changxing County, Huzhou city, Zhejiang Province China

**Keywords:** DPP4, Oral hypoglycemics, Medications, Eye, Retinopathy, Microvascular complications

## Abstract

**Background:**

The purpose of this review was to examine if dipeptidyl peptidase-4 inhibitor (DPP4i) use affects the risk of diabetic retinopathy (DR).

**Methods:**

Cohort studies published up to 20th July 2023 in the databases of PubMed, CENTRAL, Embase, Scopus, and Web of Science were searched. The adjusted effect size was pooled to calculate the odds ratio (OR).

**Results:**

Seven studies were included. Meta-analysis showed that the use of DPP4i was not associated with any significant change in the risk of DR (OR: 0.86 95% CI: 0.70, 1.06 I^2^ = 78%). The pooled analysis also found that DPP4i use was not associated with any significant risk of progression of DR (OR: 0.87 95% CI: 0.47, 1.59 I^2^ = 86%). The results did not change during sensitivity analysis.

**Conclusion:**

Present evidence from a limited number of real-world studies shows that DPP4i may not affect the incidence and progression of DR. There is a need for further studies from different countries using accurate definitions of DR and its progression to validate the current results.

**Supplementary Information:**

The online version contains supplementary material available at 10.1186/s12886-024-03535-1.

## Introduction

Diabetic retinopathy (DR) is a widely prevalent microvascular complication of diabetes mellitus affecting around 30–40% of all patients. About 100 million diabetic individuals are suffering from DR around the globe causing it to be the leading cause of visual impairment and blindness principally in the middle-aged and elderly population [1, 2]. The pathophysiology is based on hyperglycemia and altered metabolic pathway which cause oxidative stress and neurodegenerative changes in the early stage of DR. There is disruption of the blood-retinal barrier with leakage of inflammatory cytokines causing the development of microaneurysms and dot intraretinal hemorrhage which are early markers of the disease [3]. In the later stages, there is severe hypoxia which causes abnormal growth of blood vessels, macular edema, vitreous hemorrhage, and retinal detachment leading to loss of vision [4]. Important risk factors of DR include a longer duration of DM, inadequate diabetes control, and hypertension. Higher glycated hemoglobin levels strongly correlate with the progression of DR and intensive glycemic control has been associated with lower incidence and progression of the complication [3, 5]. Type 2 DM are commonly treated with oral hypoglycemic agents and considering the wide class of these drugs, it is essential to understand their effects on DR.

Dipeptidyl peptidase-4 inhibitors (DPP4i) are a class of oral hypoglycemics that act by inhibiting the DPP-4 enzyme involved in the catabolism of incretin hormone [6]. This causes increased levels of incretin which in turn reduces glucagon and increases insulin secretion [7]. FDA-approved drugs of this class include sitagliptin, saxagliptin, linagliptin, and alogliptin. These drugs have been widely used as second-line therapy in DM due to their favorable profile with minimal risk of weight gain and hypoglycemic episodes. Research suggests that DPP4i may be protective against cardiovascular events in diabetics [8]. However, mixed results have been noted on the effect of DPP4i on microvascular complications with limited data on DR [9]. Tang et al [10] in a meta-analysis of randomized controlled trials (RCTs) have shown that DPP4i may be associated with an increased risk of DR events. However, such trials have restricted inclusion criteria and these may not represent outcomes in the real world [11]. Therefore, we performed a systematic review of real-world studies to assess if DPP4i affects the risk of DR.

## Materials and methods

### Inclusion criteria

Protocol registration was done on PROSPERO and the review was allotted the number CRD42023443942. Studies were included in the review provided they: (1) were cohort in design (either retrospective or prospective) conducted on a population of type 2 DM (2) reported the association between DPP4i use or progression or risk of DR. (3) reported the association using adjusted effect size. (4) published in the English language. There was no limitation on drugs used in the comparative group. DPP4i could have been monotherapy or add-on therapy for inclusion in the review.

Studies not providing data specific to DPP4i were excluded. Randomized controlled trials, cross-sectional studies, duplicate studies, and those not reporting adjusted outcomes were also not eligible. In case articles reported overlapping data, the study with the maximum number of participants was eligible.

### Search source and strategy

Studies for the review were identified by a literature search conducted on PubMed, CENTRAL, Embase, Scopus, and Web of Science. Two reviewers were involved and the search ended on 20th July 2023. To include gray literature, a separate search was conducted on Google Scholar. Also, the bibliography of the final included studies were hand-searched for any missed articles.

Keywords used were “dipeptidyl peptidase-4 inhibitors”, “DPP4”, “DPP4i”, “retinopathy”, “microvascular complications”, “hypoglycemics”, AND “diabetes”. Different search queries were formulated using “AND” and “OR” (Supplementary Table 1). These were also replicated across the different databases.

Two investigators separately examined the titles and abstracts of searched studies after electronic deduplication. Studies relevant to the review were identified while non-relevant articles were excluded. Selected studies underwent full-text analysis against the inclusion criteria. All discords between reviewers were solved by discussion.

### Extracted data and risk of bias analysis

Two reviewers independently extracted relevant information from the studies which included: the name of the first author, publication year, region of the study, sample size, study type, included patients, use of propensity score matching, sample size, mean age, gender, DM duration, glycated hemoglobin levels, drugs used in DPP4i and control group, DR diagnosis, evaluation of DR progression, effect size of the association, and follow-up. DR was not predefined for the review and all methods of assessment and diagnosis were eligible. Study details were then cross-matched and any discrepancies were resolved in discussion with the third author.

Two reviewers assessed the methodological quality of the observational studies by the Newcastle Ottawa Scale (NOS) [12]. Points were awarded for representativeness of the study cohort, comparability of groups, and measurement of outcomes.

### Statistical analysis

PRIMA reporting guidelines were followed [13]. The meta-analysis was done on “Review Manager” (RevMan, version 5.3). Effect size data were extracted and entered into the software to derive pooled odds ratio (OR) with 95% confidence intervals (CI) of the association. Results were presented in the form of a forest plot. A random-effects model was preferred owing to methodological differences among the studies. Outliners were assessed using a sensitivity analysis involving the removal of one study at a time. Data was then presented in tabular format. A separate analysis was done for DR incidence and DR progression. Publication bias was checked with funnel plots. The chi-square-based Q statistics and I^2^ statistic was used for inter-study heterogeneity. A *p*-value of < 0.10 for Q statistic and I^2^ > 50% meant substantial heterogeneity.

## Results

**Fig. 1:**
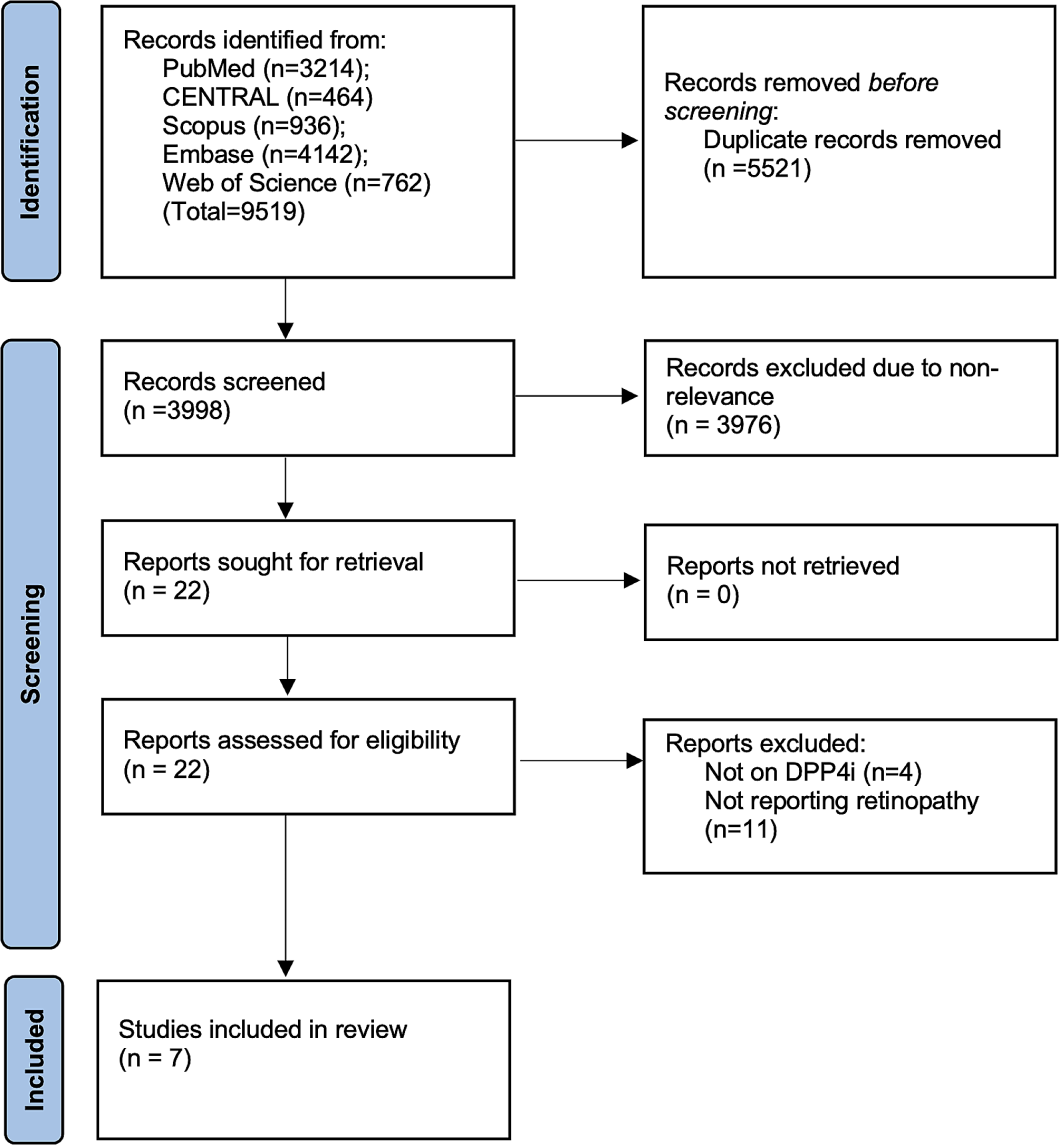
Study flowchart

The entire literature search revealed 9519 articles (Fig. 1). After the removal of duplicates, 3998 studies remained. These underwent screening by the study investigators and 22 were chosen for complete text analysis. Based on the eligibility criteria, seven were selected for inclusion [14–20].

The extracted data from the studies are presented in Table 1. The available cohort studies were published between the years 2016 to 2023 and were from the countries of Taiwan, Korea, Germany, and the USA. All were retrospective in design and four studies used propensity score matching for DPP4i users and non-users. The study of Chung et al [20] was of small sample size while the remaining included more than 3000 patients in the study and control groups. The duration of DM varied from three to eight years in the studies. Only two studies reported baseline glycated hemoglobin levels of DPP4i users and non-users. The drugs in the control group varied and mainly included sulfonylurea and metformin. The majority of studies used international classification of disease codes to identify patients with DR. Four studies reported on DR incidence while the remaining assessed progression of DR with the use of DPP4i. Follow-up of the studies varied from two to ten years. The studies had a NOS score of 6 to 9.

**Table 1:** Details of included studies

First author, year, reference number	Location	Study type	Included patients	Groups	Sample size	Mean age (years)	Male gender (%)	T2D duration (years)	HbA1c level (%)	DPP4i types	Control group drugs	DR diagnosis	Definition of DR progression	Effect size	Follow-up	NOS score
Chung 2016 [20]	Korea	R*	Age ≥ 27 years at onset of T2D and no requirement for continuous insulin treatment within 1 year of diagnosis	DPP4i Control	24 24	57.9 58.6	67 33	8 8.5	8.7 8.5	Sitagliptin, Vildagliptin, Saxagliptin, Linagliptin, Gemigliptin	SU, metformin	ETDRS scale	Increase of one or more steps on the ETDRS severity scale	0.20 (0.06, 0.69)	26.1 months	8
Kolaczynski 2016 [15]	Germany	R*	T2D aged ≥ 40 years treated with either DPP4i or SU for at least 6 months	DPP4i Control	3015 3015	64.6 63.7	57.4 54.4	3.1 3.2	7.6 7.6	Vildagliptin	SU	ICD codes	NR	0.55 (0.39, 0.77)	2.2–2.8 years	8
Kim 2018 [18]	Korea	R*	T2D aged ≥ 20 years treated with oral hypoglycemics	DPP4i Control	14,552 14,552	NR	55 55	4.6 4.4	NR	NR	SU, TZD, metformin	ICD codes for vitreous haemorrhage, blindness, or a procedure involving vitrectomy or photocoagulation; or use of an intravitreal agent	NR	1.08 (0.93, 1.26)	28.4 months	8
Wang 2018 [19]	USA	R	T2D aged ≥ 20 years treated with oral hypoglycemics for 1 year	DPP4i Control	39,292 87,073	76.5 76.7	39.3 40.9	NR	NR	NR	SU	ICD codes for DR requiring treatment	NR	0.91 (0.79, 1.04)	3 years	6
Chung 2019 [16]	Korea	R	T2D treated with metformin and receiving add-on therapy with DPP4i or SU for ≥ 90 days	DPP4i Control	6136 4447	58.1 61.1	47.7 46.4	NR	NR	NR	SU	ICD codes	Requiring intervention for DR	0.92 (0.64, 1.32)	NR	6
Kang 2021 [14]	Taiwan	R*	T2D aged ≥ 40 years treated with oral hypoglycemics	DPP4i Control	20,444 20,444	66.7 66.7	43.7 44.1	6 6	NR	NR	All types	ICD codes	Requiring intervention for DR	1.40 (1.26, 1.55)	2.5 years	8
Jensen 2023 [17]	Denmark	R	T2D treated with second line hypoglycemics	DPP4i Control	8953 1136	63 62	60.1 60.4	4 3	NR	Sitagliptin, Vildagliptin, Saxagliptin, Alogliptin Linagliptin	SU, Metformin	ICD codes	NR	0.85 (0.68, 1.06)	10	9

ETDRS, Early Treatment Diabetic Retinopathy Study; T2D, type 2 diabetes; DPP4i, Dipeptidyl peptidase-4 inhibitors; DR, diabetic retinopathy; SU, sulfonylurea; ICD, international classification of diseases; TZD, thiazolidinedione; R, retrospective

*propensity score matched

**Fig. 2:**
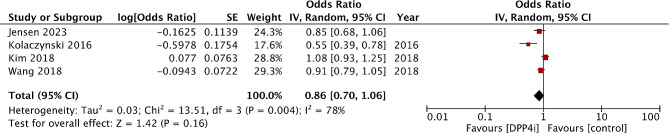
Meta-analysis of DPP4i use and DR incidence

Four studies reported data on the incidence of DR with the use of DPP4i. Meta-analysis showed that the use of DPP4i was not associated with any significant change in the risk of DR (OR: 0.86 95% CI: 0.70, 1.06 I^2^ = 78%) (Fig. 2). The exclusion of any study did not change the significance of the results.

**Fig. 3:**
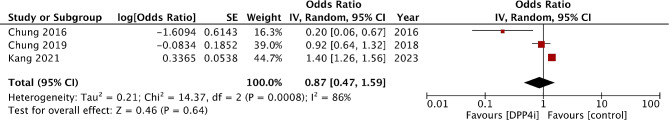
Meta-analysis of DPP4i use and DR progression

Three studies reported data on the progression of DR with the use of DPP4i. Meta-analysis showed that DPP4i use was not associated with any significant risk of progression of DR (OR: 0.87 95% CI: 0.47, 1.59 I^2^ = 86%) (Fig. 3). The results did not change on sensitivity analysis.

## Discussion

Management of DM by oral hypoglycemic agents has had a significant impact on glycemic control and prevention of micro and macrovascular complications of DM. Sulfonylureas and metformin have been traditionally the first line of oral hypoglycemics, however, a large number of newer drugs including DPP4i, glucagon-like peptide-1 receptor agonists (GLP-1a), sodium-glucose cotransporter-2 inhibitors (SGLT2i), thiazolidinediones, and alpha-glucosidase inhibitors have been developed in recent times [21]. These drugs can be used in monotherapy or with traditional first-line agents for the management of DM. Indeed, it has been well-established that good glycemic control is essential in reducing the risk of microvascular complications of DM, including DR [22]. While the newer oral hypoglycemic agents act to reduce blood sugar levels, they all have different mechanisms of action which may alter the risk of diabetic complications. Xie et al [23] have shown that DM patients treated with SGLT2i or GLP1a have a reduced risk of adverse kidney outcomes as compared to those treated with DPP4i or sulfonylureas. Lin et al [24] in a meta-analysis have demonstrated a slightly increased risk of lower limb amputations in patients treated with canagliflozin. Recently, Ma et al [25] in a pooled analysis of RCTs have shown that the use of SLGT2i did not affect the risk of DR.

In this review, we combined data from seven studies to examine the risk of DR with the use of DPP4i. The limited data available demonstrated that the use of DPP4i did not increase the risk of new DR in diabetic patients. Out of the four studies included in the meta-analysis, only one study by Kolaczynski et al [15] reported a significantly reduced risk of DR with DPP4i (Vildagliptin) as compared to sulfonylureas while all others reported no such significant difference. Important to note is that this study was industry-funded and may be prone to bias. For DR progression, a pooled analysis of three studies demonstrated no significant impact of DPP4i use on the complication. The results were not coherent among studies with one small study by Chung et al [20] showing a reduced risk of DR progression and a large study by Kang et al [14] demonstrating an increased risk of DR progression. The last remaining study by Chung et al [16] found no effect of DPP4i on the progression of DR. In addition to variation in sample size, the differences in the study populations and the method of assessing DR progression could have led to variable results. Chung et al [16] and Kang et al [14] used ICD codes for DR interventions to assess DR progression while the small study of Chung et al [20] used a validated scale for the same.

The lack of difference in DR incidence and progression noted in real-world studies has been replicated in the small number of RCTs assessing this outcome. In the TECOS trial comparing sitagliptin with placebo, the risk of DR was 2.8% in the sitagliptin group and 2.2% in the control group [26]. The CARMELINA trial assessed the efficacy of linagliptin vs. placebo for type 2 DM. One of the outcomes was the composite ocular endpoint defined as the need for intervention for diabetic retinopathy or vitreous hemorrhage or diabetes-related blindness. The trial reported no difference between the study (1%) and control groups (1.4%) [27]. Ott et al [28] conducted a cross-over RCT comparing saxagliptin with placebo for six weeks in 50 patients. 43 patients were finally assessed for retinal arteriolar structure and retinal capillary flow by scanning laser Doppler flowmetry. The study found that saxagliptin results in the normalization of retinal blood flow with improvement in central hemodynamics.

Similar contrasting evidence has been reported in animal and in-vitro studies examining the effect of DPP4i on retinal cells. Goncalves et al [29] in an animal study have shown that DPP4i reduces inflammation and apoptosis in retinal cells thereby exerting a protective effect on the blood-retinal barrier integrity. The same authors have also shown that sitagliptin has an antioxidative effect with a positive modulatory action on the vascularity of retinal endothelial cells [30]. Kolibabka et al [31] in another animal study found that linagliptin demonstrates an anti-angiogenic effect in mice with oxygen-induced retinopathy which could be protective against DR in humans. Contrastingly, a recent study published in 2020 has found that long-term exposure to DPP4i may weaken the blood-retina barrier and may induce retinal edema [32]. Another study has noted that DPP4i can cause separation of the endothelial cellular junction by collection of stromal cell-derived factor 1α and phosphorylating vascular endothelial cadherin leading to an increase in retinal vascular permeability [33].

Given the current data, the effects of DPP4i on the retina are still unclear and conflicting both in the experimental and clinical setup. Clinically, a number of factors could explain such variations as the duration of diabetes, baseline glycemic control, use of other hypoglycemic agents, duration of therapy, etc. While RCTs have a low risk of bias due to the predefined inclusion criteria, blinding, and prospective nature of the study; the study population tends to be restrictive and may not reflect the complex scenario seen in the real world [11]. The current review is the first meta-analysis to collate data from real-world studies to examine the impact of DPP4i on DR. A thorough literature search was carried out and a separate analysis was conducted for DR incidence and progression.

Nevertheless, drawbacks of the review include the observational nature of the data used in the studies which is derived from medical records. The possibility of selection bias in the prescription of drugs cannot be ruled out. Duration and compliance of drug therapy cannot be firmly confirmed as data is derived mostly from pharmacy records. Furthermore, there were some variations in the drugs used in the control group with some studies using newer second-line oral hypoglycemics as well. The follow-up duration of most studies was about two years and it is still unclear how DPP4i affects DR in the long-term. Diagnosis of DR and its progression was not clinical and was sourced from medical records or the requirement of intervention for DR. Such a definition may not be completely inclusive of patients with DR progression. One study [20] used the ‘Early Treatment Diabetic Retinopathy Study’ scale while all other studies used ICD codes to identify DR. However, the results did not change on sensitivity analysis. Further, due to lack of data, it was not possible to examine the effect of different types of DPP4i on DR incidence and progression. Also important to note is that several risk factors can affect the incidence and progression of DR. Research suggests that older age, duration of DM, hypertension, glucose levels, lipid profile, hypoglycemic drugs, and other diabetic microvascular complications are important confounders for the risk of DR [1–4]. While we used only adjusted data from the included studies, it is possible that several known and unknown confounders could have been missed which could have affected outcomes. Moreover, there were two studies from the same country in our meta-analysis, with some overlap of study duration [16, 20]. Therefore, there may be a possibility of partial overlap between the studies. Lastly, the effect size mentioned in Table 1 for each study could not be exactly replicated in the meta-analysis software as Review Manager auto-calculates the upper end of the 95% CI when lower end of 95% CI is entered in the software. This leads to minor uncorrectable variations for the upper end of 95% CI.

## Conclusions

Present evidence from a limited number of real-world studies shows that DPP4i may not affect the incidence and progression of DR. There is a need for further studies from different countries using accurate definitions of DR and its progression to validate the current results.

### Electronic supplementary material

Below is the link to the electronic supplementary material.

Supplementary Material 1

Supplementary Material 2

## Data Availability

No datasets were generated or analysed during the current study.
