# Supplementary material for: Association between dipeptidyl peptidase-4 inhibitor use and diabetic retinopathy: a systematic review and meta-analysis of real-world studies
Source: BMC Ophthalmol. 2024 Jun 28;24:272. doi: 10.1186/s12886-024-03535-1 (PMC11212248; doi:10.1186/s12886-024-03535-1)
Supplement: Supplementary file 2 — Supplementary Material 2 [file 12886_2024_3535_MOESM2_ESM.docx]

Supplementary Table 1: Search strategy

| Key point | Details |
| --- | --- |
| ((((dipeptidyl peptidase-4 inhibitors) OR (DPP4)) OR (DPP4i)) AND (microvascular complications)) AND (diabetes) | ("dipeptidyl peptidase iv inhibitors"[Pharmacological Action] OR "dipeptidyl peptidase iv inhibitors"[Supplementary Concept] OR "dipeptidyl peptidase iv inhibitors"[All Fields] OR "dipeptidyl peptidase 4 inhibitors"[All Fields] OR "dipeptidyl peptidase iv inhibitors"[MeSH Terms] OR ("dipeptidyl peptidase"[All Fields] AND "iv"[All Fields] AND "inhibitors"[All Fields]) OR ("dpp4 protein human"[Supplementary Concept] OR "dpp4 protein human"[All Fields] OR "dpp4"[All Fields]) OR ("dpp4i"[All Fields] OR "dpp4is"[All Fields])) AND (("microvascular"[All Fields] OR "microvascularity"[All Fields] OR "microvascularization"[All Fields] OR "microvascularized"[All Fields]) AND ("complicances"[All Fields] OR "complicate"[All Fields] OR "complicated"[All Fields] OR "complicates"[All Fields] OR "complicating"[All Fields] OR "complication"[All Fields] OR "complication s"[All Fields] OR "complications"[MeSH Subheading] OR "complications"[All Fields])) AND ("diabete"[All Fields] OR "diabetes mellitus"[MeSH Terms] OR ("diabetes"[All Fields] AND "mellitus"[All Fields]) OR "diabetes mellitus"[All Fields] OR "diabetes"[All Fields] OR "diabetes insipidus"[MeSH Terms] OR ("diabetes"[All Fields] AND "insipidus"[All Fields]) OR "diabetes insipidus"[All Fields] OR "diabetic"[All Fields] OR "diabetics"[All Fields] OR "diabets"[All Fields]) |
| ((DPP4) OR (DPP4i)) AND (retinopathy) | ("dpp4 protein human"[Supplementary Concept] OR "dpp4 protein human"[All Fields] OR "dpp4"[All Fields] OR ("dpp4i"[All Fields] OR "dpp4is"[All Fields])) AND ("retinal diseases"[MeSH Terms] OR ("retinal"[All Fields] AND "diseases"[All Fields]) OR "retinal diseases"[All Fields] OR "retinopathies"[All Fields] OR "retinopathy"[All Fields]) |
| (dipeptidyl peptidase-4 inhibitors) AND (retinopathy) | ("dipeptidyl peptidase iv inhibitors"[Pharmacological Action] OR "dipeptidyl peptidase iv inhibitors"[Supplementary Concept] OR "dipeptidyl peptidase iv inhibitors"[All Fields] OR "dipeptidyl peptidase 4 inhibitors"[All Fields] OR "dipeptidyl peptidase iv inhibitors"[MeSH Terms] OR ("dipeptidyl peptidase"[All Fields] AND "iv"[All Fields] AND "inhibitors"[All Fields])) AND ("retinal diseases"[MeSH Terms] OR ("retinal"[All Fields] AND "diseases"[All Fields]) OR "retinal diseases"[All Fields] OR "retinopathies"[All Fields] OR "retinopathy"[All Fields]) |
| ((hypoglycemics) AND (diabetes)) AND (retinopathy) | ("hypoglycaemics"[All Fields] OR "hypoglycemic agents"[Pharmacological Action] OR "hypoglycemic agents"[Supplementary Concept] OR "hypoglycemic agents"[All Fields] OR "hypoglycaemic"[All Fields] OR "hypoglycemic agents"[MeSH Terms] OR ("hypoglycemic"[All Fields] AND "agents"[All Fields]) OR "hypoglycemic"[All Fields] OR "hypoglycemics"[All Fields]) AND ("diabete"[All Fields] OR "diabetes mellitus"[MeSH Terms] OR ("diabetes"[All Fields] AND "mellitus"[All Fields]) OR "diabetes mellitus"[All Fields] OR "diabetes"[All Fields] OR "diabetes insipidus"[MeSH Terms] OR ("diabetes"[All Fields] AND "insipidus"[All Fields]) OR "diabetes insipidus"[All Fields] OR "diabetic"[All Fields] OR "diabetics"[All Fields] OR "diabets"[All Fields]) AND ("retinal diseases"[MeSH Terms] OR ("retinal"[All Fields] AND "diseases"[All Fields]) OR "retinal diseases"[All Fields] OR "retinopathies"[All Fields] OR "retinopathy"[All Fields]) |
